# 30315 Successes in the COVID-19 Era: Novel Peer-Mentoring Series for Junior and Mid-Career Academic Faculty Across a University Campus

**DOI:** 10.1017/cts.2021.566

**Published:** 2021-03-30

**Authors:** Lauren Chernick, Teresa Lee, Stephanie Lovinsky-Desir, Marisa Span, Gissette Reyes-Soffern, Jennifer Woo Baidal, Anne L. Taylor, Daichi Shimbo, Clara Lapiner, Brett Anderson

**Affiliations:** Columbia University Irving Medical Center

## Abstract

ABSTRACT IMPACT: Partnering with academic offices to promote peer-mentoring in a virtual format is feasible, novel, and well-received across a major academic campus. Particularly during a pandemic, the success of this programmatic effort highlights the continued need for peer-to-peer support. OBJECTIVES/GOALS: To identify feasibility and key lessons learned from the planning and implementation of a virtual, interdisciplinary group peer-mentoring series, implemented broadly across an academic medical center in New York City. METHODS/STUDY POPULATION: ASPIRE! (Accountability & Safe-Space to Promote, Inspire, Recharge, & Empower one another!) is a group of seven interdisciplinary mid-career academic women faculty, who began collaborations as CTSA KL2 scholars. Our mission is to support interdisciplinary peer coaching for advancement of gender and racial equity among academic faculty and leaders. We designed and implemented a series of virtual symposia focused on essential struggles for clinicians and investigators at during the COVID-19 pandemic. Partnering with Columbia’s CTSA, Office for Women and Diverse Faculty, and Office for Research, we invited leaders in psychiatry/psychology, early childhood education, organization/team management, and academic advancement as keynote speakers and facilitated peer-mentoring virtual breakouts. RESULTS/ANTICIPATED RESULTS: These efforts resulted in the completion of four separate 1.5-hour symposia, each with keynote speakers, discussions with academic leaders, and 30-minute breakout peer-mentoring sessions. Session topics included Calibrating Expectations, Helping Families Thrive, Managing Remote Teams, and Faces and Phases of Stress. Enrollment ranged from 30 to 70 participants per session. Participants reported: (1) Keynotes focused on actionable solutions stimulated the most productive conversations; (2) Peers from different disciplines and career stages provided a range of actionable recommendations tested within local contexts; (3) The greatest learning came from the peer-to-peer breakout group sessions. DISCUSSION/SIGNIFICANCE OF FINDINGS: Partnering with academic offices to promote interdisciplinary, peer-mentoring in a virtual format is feasible, novel, and can be well-received across a major academic campus during the COVID-19 pandemic. The success of this programmatic effort highlights the continued need for expanded peer-to-peer support in academia.

